# Elevated breast cancer mortality among highly educated Asian American women

**DOI:** 10.1371/journal.pone.0268617

**Published:** 2022-05-18

**Authors:** Heidy N. Medina, Karen E. Callahan, Tulay Koru-Sengul, Sfurti Maheshwari, Qinran Liu, Neha Goel, Paulo S. Pinheiro

**Affiliations:** 1 Department of Public Health Sciences, University of Miami Miller School of Medicine, Miami, Florida, United States of America; 2 Department of Environmental and Occupational Health, School of Public Health, University of Nevada Las Vegas, Las Vegas, Nevada, United States of America; 3 Sylvester Comprehensive Cancer Center, Department of Public Health Sciences, University of Miami Miller School of Medicine, Miami, Florida, United States of America; 4 Division of Surgical Oncology, Department of Surgery, University of Miami Miller School of Medicine, Miami, Florida, United States of America; The University of Mississippi Medical Center, UNITED STATES

## Abstract

**Background:**

Postmenopausal breast cancer (PMBC) is the most commonly diagnosed and the second leading cause of cancer death among women in the US. Research examining the association between PMBC and education level has been inconsistent; no study in the US has examined how educational level impacts PMBC mortality in Asian American women, a largely immigrant population with above-average educational attainment.

**Methods:**

California Vital Statistics data from 2012–2017 were analyzed to derive age-adjusted mortality rate ratios (MRRs) by education level (associates degree or above referred to as “higher education”, high school, less than high school) and race [Non-Hispanic White (NHW), Asian/Pacific Islander (Asian), and its two largest subpopulations: Chinese and Filipino] from negative binomial regression models.

**Results:**

PMBC mortality for both NHWs and Asians was greater among women with higher education compared to those who did not complete high school: NHWs had 22% higher PMBC mortality (MRR 1.22; 95% CI: 1.14–1.31) and Asians had 2.6 times greater PMBC mortality (MRR 2.64; 95% CI: 2.32–3.00) than their counterparts who did not complete high school. Asians in the lowest education level had 70% lower mortality than NHWs (MRR 0.30; 95% CI: 0.27–0.34). This mortality advantage among Asians was greatly reduced to only 27% lower among the highest educated (MRR 0.73; 95% CI: 0.68–0.78). For higher educated Filipina women, no mortality advantage was evident compared to NHWs (MRR 0.96; 95% CI: 0.88–1.05).

**Conclusion:**

PMBC mortality for higher educated Asian women is elevated in comparison to their counterparts with less education. Given that PMBC survival is greater among those with higher education, our findings strongly suggest an excess in the incidence of PMBC (more than double) among higher educated Asian women; this warrants more research into potentially modifiable causes of PMBC in this burgeoning population.

## Introduction

Breast cancer is the most commonly diagnosed cancer and the second leading cause of cancer death among all women in the United States (US) [1]. In 2021, 281,550 women in the US were estimated to be newly diagnosed with breast cancer and 43,600 deaths occurred, accounting for nearly 31% and 15% of all new cancer cases and deaths, respectively [1]. Approximately 80% to 89% of breast cancers occur after menopause [1].

Socioeconomic disparities associated with educational attainment impact incidence, survival, and mortality rates of cancer [2]. While higher education levels are associated with lower mortality rates for most cancers [3–5], increased breast cancer mortality has been associated with higher educational levels and/or socioeconomic status in some studies outside the US [6–9]. Reasons for this excess mortality have been largely attributed to differences in reproductive factors: specifically, total number of children (or nulliparity) and older maternal age at first birth [6–9]. In the US, similar studies have shown inconsistent results [2, 10–12].

The Asian American population in the US in 2019 was over 20 million. With an increase of 81% since 2000, Asians are the fastest growing minority group in the US [13]. Generally, Asians demonstrate a broad range of favorable health indicators including relatively low cancer incidence and mortality and high survival [14–20]. In the US, nearly 71% of adult Asians are foreign-born immigrants comprising a highly heterogeneous population with varying degrees of acculturation, educational attainment, and socioeconomic status [13]. However, Asians overall have higher annual household incomes, lower levels of poverty, and higher levels of education than the average American [13]. Notably, in contrast to most other racial/ethnic groups in the US, breast cancer incidence among Asians has been increasing by a gradual 0.8% annually since 1992 [16] and mortality rates have not been decreasing [21].

Despite this unique context, the interplay between educational attainment and cancer mortality has received little attention; no study thus far has looked at how educational level impacts breast cancer mortality rates among Asian American women.

California is home to nearly a third of all Asian Americans in the US [22], providing a distinctive opportunity for the population-based examination of breast cancer in Asian Americans and the larger subpopulations including Chinese and Filipinos. In this study, we examine patterns of postmenopausal breast cancer (PMBC) mortality according to different levels of educational attainment for Asians in California while characterizing racial differences between Asians and the reference population, non-Hispanic Whites.

## Methods

Cancer mortality data from 2012–2017 were obtained from the California Department of Public Health. Variables examined included age, sex, race/ethnicity, birthplace, educational attainment, and underlying cause of death. Women whose primary cause of death was breast cancer, inclusive of International Classification of Diseases, Tenth Revision (ICD-10) codes C50.0-C50.9, were included. Only women ages 50 and above were included to approximate postmenopausal cases.

Cancer mortality was examined for the Non-Hispanic White (NHW) population and for Asian/Pacific Islanders (hereafter referred to as Asians) in aggregate, as well as for the two largest Asian subpopulations, Chinese and Filipino. The aggregate Asian group included all decedents of Asian descent, including not only Chinese and Filipino but also South Asian, Vietnamese, Korean, Japanese, Southeast Asian, and Native Hawaiian/Other Pacific Islander decedents. Educational attainment was available for 99% of decedents and classified into three categories: less than high school education, high school completion or equivalent, and Associate’s degree or higher (hereafter also referred to as “higher education”).

Population denominators by race, age, and education level (as defined above, same as for PMBC deaths) for the state of California were obtained from the American Community Survey for each single year from 2012–2017 and then pooled [22] (Table 1). Age-adjusted mortality rates (AAMRs) for cancers for this six-year period were annualized, age-standardized to the 2000 US Standard Population, and expressed per 100,000 persons. Truncated AAMRs were calculated to only include those above age 50. We calculated 95% confidence intervals for AAMRs using Tiwari’s gamma intervals modification method [23]. To directly compare the mortality rates, we computed age-adjusted mortality rate ratios (MRRs) and corresponding 95% confidence intervals from negative binomial regression models to account for the different age structures of each population [24]. Ratios from multivariable models that included race, age group (50–64, 65–79, 80+), educational level, and the interaction term between race and educational level were studied (Table 2). Since the interaction term was statistically significant, MRRs comparing educational levels were stratified by race (Table 3), using the lowest education level (less than high school) as the reference group. MRRs comparing race were also stratified by educational level (Table 3), using NHWs as the reference group.

**Table 1:** Population at risk and characteristics of NHW and Asian female decedents from PMBC in California (CA), 2012–2017.

	NHW n (%)	Asian		
		All Combined^a^ n (%)	Chinese n (%)	Filipino n (%)
**Population Data, CA 2012-2017** ^**b**^				
**Annualized Population (Total)**	3,386,344	1,092,895	307,966	280,345
**Person-years Accumulated by Education Level**				
Less than High School	1,311,414 (6.5%)	1,501,404 (22.9%)	504,306 (27.3%)	199,860 (11.9%)
High School or Equivalent	9,969,264 (49.1%)	2,194,104 (33.5%)	546,552 (29.6%)	544,032 (32.3%)
Associate’s Degree or Higher	9,037,386 (44.5%)	2,861,862 (43.6%)	796,938 (43.1%)	938,178 (55.8%)
**Cancer Deaths, CA 2012-2017** ^**b**^				
**Total Deaths**	15,095 (86.4%)	2372 (13.6%)	611 (25.8%)	809 (34.1%)
**Education Level**				
Less than High School	998 (6.6%)	305 (12.9%)	109 (17.8%)	54 (6.7%)
High School or Equivalent	8384 (55.5%)	893 (37.6%)	210 (34.4%)	229 (28.3%)
Associate’s Degree or Higher	5713 (37.8%)	1174 (49.5%)	292 (47.8%)	526 (65.0%)
**Age at Death**				
50–64	4388 (29.1%)	1075 (45.3%)	283 (46.3%)	372 (46.0%)
65–79	5626 (37.3%)	804 (33.9%)	178 (29.1%)	313 (38.7%)
80+	5081 (33.7%)	493 (20.8%)	150 (24.5%)	124 (15.3%)
**AAMR**^**c**^ **per 100,000 (95% CI)**				
Less than High School	58.4 (54.2–62.8)	17.8 (15.8–20.1)	17.3 (13.9–21.3)	23.7 (16.4–32.9)
High School or Equivalent	75.0 (73.3–76.7)	40.6 (37.9–43.4)	38.2 (33.1–43.9)	41.9 (36.6–47.8)
Associate’s Degree or Higher	66.8 (65.1–68.7)	46.1 (43.3–49.0)	43.4 (38.1–49.3)	60.0 (54.6–65.8)

Abbreviation: AAMR, Age-adjusted mortality rate; NHW, Non-Hispanic White; PMBC, Postmenopausal breast cancer.

a. Includes Chinese and Filipino and all other groups not detailed here (e.g., Japanese, Korean, etc.)

b. includes females over age 50

c. Age-adjusted to the 2000 US Standard Population and truncated for age-groups above age 50.

**Table 2:** PMBC MRRs^a^ for NHW & all Asian females in California, 2012–2017.

	Model 1	Model 2	Model 3
	MRR (95% CI)	MRR (95% CI)	MRR (95% CI)
**Race**			
NHW	Referent	Referent	Referent
Asian (all combined)^b^	0.52 (0.42–0.65)	0.50 (0.42–0.59)	0.31 (0.23–0.40)
**Age**			
50–64	Referent	Referent	Referent
65–79	1.72 (1.35–2.20)	1.72 (1.41–2.10)	1.73 (1.46–2.04)
80+	3.17 (2.41–4.16)	3.15 (2.53–3.93)	3.21 (2.66–3.86)
**Education Level**			
Less than High School	--	Referent	Referent
High School or Equivalent	--	1.65 (1.33–2.04)	1.29 (1.01–1.65)
Associate’s Degree or Higher	--	1.67 (1.35–2.07)	1.15 (0.90–1.47)
Race*Education Level Interaction			
NHW*Less than High School	--	--	Referent
Asian (all combined)* High School or Equivalent	--	--	1.78 (1.23–2.55)
Asian (all combined)*Associate’s Degree or Higher	--	--	2.26 (1.57–3.25)

Abbreviation: MRR, Mortality rate ratio; NHW, Non-Hispanic White; PMBC, Postmenopausal breast cancer.

a. MRRs derived from negative binomial regression

b. Includes Chinese and Filipino and all other groups not detailed here (e.g., Japanese, Korean, etc.).

**Table 3:** PMBC MRRs^a^ by level of education and race, California, 2012–2017.

	MRR (95% CI)				
	Education Level (referent = Less than High School)				
**Race**	High School or Equivalent		Associate’s Degree or Higher		
NHW	1.37 (1.29–1.47)		1.22 (1.14–1.31)		
Asian (all combined)^b^	2.33 (2.04–2.65)		2.64 (2.32–3.00)		
Chinese	2.20 (1.74–2.78)		2.43 (1.93–3.06)		
Filipino	1.95 (1.44–2.64)		2.77 (2.07–3.69)		
	**Race** (referent = NHW)			
**Education Level**	Asian (all combined)^b^	Chinese		Filipino
Less than High School	0.30 (0.27–0.34)	0.31 (0.26–0.38)		0.36 (0.27–0.47)
High School or Equivalent	0.55 (0.51–0.59)	0.53 (0.46–0.60)		0.57 (0.49–0.67)
Associate’s Degree or Higher	0.73 (0.68–0.78)	0.67 (0.59–0.75)		0.96 (0.88–1.05)

Abbreviation: MRR, Mortality rate ratio; NHW, Non-Hispanic White; PMBC, Postmenopausal breast cancer.

a. MRRs derived from negative binomial regression

b. Includes Chinese and Filipino and all other groups not detailed here (e.g., Japanese, Korean, etc.).

Lastly, prevalence data by educational level for known breast cancer risk factors—parity, hormone replacement therapy (HRT), and obesity—as well as for mammography screening were obtained for women aged 40 and older from the 2012–2017 National Health Interview Surveys (NHIS) [25]. We chose the age cut-off of 40 years in order to attain an understanding of exposure to PMBC risk factors of longer duration such as obesity, and it is also the age recommended to initiate mammography screening [26]. The average prevalence across all available survey years was computed for NHWs and Asians to help interpret findings from the mortality analysis (S1 Table) [25]. NHIS data for the West region was weighted using the final sample adult weights to incorporate complex survey design and provide nationally representative estimates. The *P* for linear trend tests tested the null hypothesis that the slope of linear regression (prevalence of PMBC risk factors as outcome and education level as the predictor) was 0. All hypothesis testing was two-sided and considered statistically significant at *p* < 0.05.

Statistical analysis software IBM SPSS 27 (Armonk, NY: IBM Corp) and SAS v9.4 software (SAS Institute Inc., Cary, NC, USA) were used for data management and analyses. This study is the result of a secondary data analysis with deidentified data. The data was therefore fully anonymized and informed consent was waived. The study is covered under California Department of Public Health Protocol ID #15-08-2161, PI: PS Pinheiro.

## Results

### Study population

A total of 17,467 deaths due to PMBC from the six-year period between 2012–2017 in California were examined: NHW women accounted for 86% (n = 15,095) of all deaths and Asians for 14% (n = 2,372) (Table 1). Among Asian decedents, Chinese and Filipinas accounted for 26% and 34% of PMBC deaths, respectively. Across all Asian groups and in the aggregate Asian group, the greatest proportion of the population at risk and of PMBC deaths occurred among those with the highest educational attainment; however, for NHWs, a larger at-risk population (49%) and greater proportion of deaths (56%) were observed among those with a high school or equivalent degree.

### PMBC mortality rates

Across all levels of education, PMBC AAMRs per 100,000 were considerably lower among Asian decedents than NHW women, especially for Chinese women (Table 1). The highest AAMR among NHWs was observed among those with a high school degree or equivalent (75.0; 95% CI: 73.3–76.7); among Asians the highest was at the Associate’s degree or higher level (46.1; 95% CI: 43.3–49.0). AAMRs per 100,000 were consistently higher among Filipinas than Chinese females: 23.7, 41.9, and 60.0 for Chinese women compared to 17.3, 38.2 and 43.4 for Filipinas at the lowest, middle, and highest level of educational attainment, respectively.

### PMBC mortality rate ratios

In multivariable analysis, Asian decedents had 48% (MRR 0.52; 95% CI:0.42–0.65) lower mortality than their NHW counterparts after adjustment for age (Table 2, Model 1). Those with an associate degree or higher had a 67% greater mortality (MRR 1.67; 95% CI: 1.35–2.07) than those with less than a high school education (Table 2, Model 2) after adjusting for differences in age and race. However, a significant interaction was observed between race and educational level (*p<0*.*001*) (Table 2, Model 3). Thus, to better understand the joint effect of race and education from this significant interaction term, we examined MRRs stratified first by race and then by level of education (Table 3).

### PMBC MRRs stratified by level of education and by race

For NHW women, PMBC mortality was highest for those with a high school degree (the middle level of educational attainment), 37% higher than those with less than a high school education (MRR 1.37; 95% CI: 1.29–1.47); those with higher education had 22% higher mortality (MRR 1.22; 95% CI: 1.14–1.31). However, PMBC mortality for Asians followed an educational gradient. Compared to those with less than high school education, PMBC mortality among Asians was 164% (MRR 2.64; 95% CI: 2.32–3.00) and 133% (MRR 2.33; 95% CI: 2.04–2.65) greater for those with higher education and a high school degree, respectively. The pattern was similar for Chinese and Filipina women; higher educated Filipina and Chinese women showed significantly higher PMBC mortality, MRR 2.77 (95% CI, 2.07–3.69) and MRR 2.43 (95% CI, 1.93–3.06), respectively, than their counterparts with less than a high school education (Table 3).

PMBC mortality for Asians in aggregate and for Chinese and Filipinas were lower than their NHW counterparts across all levels of educational attainment (Table 3). Asian women with the lowest level of education had the greatest advantage, with 70% lower mortality (MRR 0.30; 95% CI: 0.27–0.34) than NHW women without a high school degree. As educational level increased, the mortality advantage decreased but remained sizable, with death rates 45% (MRR 0.55; 95% CI: 0.51–0.59) and 27% (MRR 0.73; 95% CI: 0.68–0.78) lower among Asians than their NHW counterparts with a high school degree or higher education, respectively. Notably, Filipina women at the highest level of education had PMBC mortality not significantly different than NHW women (MRR 0.96; 95% CI: 0.88–1.05).

### Prevalence of PMBC risk factors

Prevalence of several known risk factors for PMBC are shown for Asian and NHW women by educational attainment using NHIS data in S1 Table. NHW women at the higher levels of educational attainment were less likely to be obese compared to their counterparts with the lowest level of education. Meanwhile, amongst Asian women, higher prevalence in the use of HRT, mammography screening, older mean age at birth of first child, and greater nulliparity/lower parity (especially in the proportion of women with ≥3 births) is seen with increasing levels of education (all, *p for linear trend <0*.*01*).

## Discussion

To our knowledge, this is the first population-based assessment of the association between educational attainment and PMBC mortality among Asian women in the US. Our results show that both NHW and Asian postmenopausal women with higher levels of educational attainment (i.e., Associate’s degree or higher) have higher mortality rates for PMBC than women who did not complete high school. Notably, while this excessive mortality among higher educated women is relatively small among NHWs, just one-fifth higher, it is quite sizable among Asian women whose rates are over two and a half times greater than their lower educational level counterparts. These substantial differences by educational level are somewhat unexpected and suggest that potentially modifiable PMBC risk factors, more prevalent among women with higher educational attainment, especially Asian women, may be targets for intervention.

The association between education and breast cancer mortality has always been unique [3–5]. For most common cancer sites, including lung, colorectal, prostate, liver, and cervix, an inverse association exists between educational attainment and cancer mortality, i.e., lower mortality rates with higher educational attainment, have been documented among NHWs, Blacks, Hispanics, and Asians [3–5]. Conversely, for breast cancer, evidence suggests a positive association between mortality and educational level; the higher the educational level, the higher the mortality rate [6–9]. However, existing studies were primarily conducted outside the US, during earlier time periods, and included only Caucasian women. No studies of the association between breast cancer and educational attainment have focused on postmenopausal Asian women living in the US.

Mortality as a population-based outcome measure (the denominator being the total population) is a product of both incidence and survival indicators. An extensive list of risk factors which impact incidence, and prognostic factors which impact survival, has been established for breast cancer. Many of these factors associated with education level [9, 27], include reproductive factors that impact incidence, such as older age at first birth, nulliparity, lack of breastfeeding, older age at menopause, early menstruation, and use of oral contraceptives and/or HRT [28]. For survival, higher education typically has a beneficial influence on prognostic factors, such as knowledge about cancer risk, attitudes toward and access to routine mammography screening, diagnosis at earlier stages, timeliness of treatment initiation, receipt of appropriate treatments, and exposure to lifestyle and/or environmental factors that impact tumor progression [29–32].

Factors that could theoretically be responsible for the observed excess mortality among women with higher educational attainment compared to those with lower education include an unfavorable case mix of PMBC subtypes (more aggressive tumor biology in terms of estrogen receptor status, HER2 status, etc.) [33] or later stages at diagnosis. These differences would translate to lower survival for more educated women. However, the opposite has been repeatedly found [29, 34, 35]. Survival after a breast cancer diagnosis has consistently been shown to be highest among those with the highest level of education and lowest for those with the lowest educational attainment, even after taking into account factors such as use of mammography screening, stage at diagnosis, and lifestyle-related factors (e.g., age at first birth) [29, 34, 35]. Given this evidence, our findings of higher PMBC mortality on a population basis among Asian women with higher educational attainment can only be a result of a substantial and somewhat alarming higher incidence of PMBC among these Asian women.

Previous literature has shown that among US immigrant populations, increasing levels of acculturation, “learning and adopting the mainstream culture” [36] of the majority NHW population, are associated with the adoption of "westernized" behaviors/lifestyle factors such as delayed and/or reduced childbearing, sedentary lifestyle, higher BMI, and increased alcohol consumption which can affect breast cancer risk and thus impact mortality correspondingly [16, 37–40]. The role of acculturation in breast cancer risk among Asians has been the focus of several studies [41, 42], but few have examined how the process of acculturation may occur at different speeds across education levels. Based on data from NHIS, the intra-racial differences across educational levels in parity, specifically the bearing of 3 or more children, use of HRT, and mammography uptake, was more pronounced among Asian than NHW women [25] (S1 Table). A more rapid transition in the risk factor profiles of Asians in the US with higher levels of education might partially explain their higher incidence leading to the observed higher PMBC mortality in comparison to Asians with lower levels of education.

The PMBC mortality advantage seen for Asian women in comparison to NHW women is not in itself surprising, as the advantage for breast cancer in general has been well documented [14–18, 43]. However, when partitioned according to educational attainment, the advantage differed remarkably; particularly striking was how much lower PMBC mortality was among those with less than a high school education. Yet, as the level of education increased, mortality increased greatly among Asian women, approaching that of NHWs. This study also shows differences within Asians; for the two groups with populations sizable enough to study alone, Filipina women with higher educational attainment had higher PMBC mortality than their Chinese counterparts. These findings are consistent with previous studies showing Filipina women with higher overall breast cancer mortality rates than Chinese women, higher than all other Asian groups in the US in most studies [14, 16–18]. Possible explanations include more rapid acculturation [44] and/or lower survival [19]. In fact, counter to the usual finding that Asian women always have lower breast cancer mortality rates than NHW women, no mortality advantage for Filipinas in relation to NHWs was observed at the highest level of education in the current study. These results are consistent with risk factor data showing a higher degree of acculturation in this group; according to the California Health Interview Survey (CHIS), the prevalence of obesity, current smokers, alcohol use, and binge drinking is higher among Filipinas than Chinese women [45]. Moreover, while no evidence exists to suggest lower uptake of routine mammography screening among Filipina women [45, 46], they have a higher prevalence of nulliparity, HRT and hormone use, and younger age at menarche [45].

There are several strengths of this study that merit mention. While the overall lower PMBC mortality observed among Asians in relation to NHWs is consistent with past studies [14–18], to our knowledge, this is the first study to document the positive association between education and PMBC mortality amongst Asian women. The population-based nature of this study, in which every death due to PMBC in 2012–2017 was included, limits the extent of selection bias that is inherent in other study designs. California is also ideally representative, not only in terms of age structure, with the largest proportion of older-aged Asians, but also population size, as the state is home to almost one-third of the entire Asian population in the US, with 36% of Chinese and 40% of Filipinos [22]. Additionally, death certificate data is highly complete for specific race, over 99% for Asian subpopulations.

Nonetheless, this study is subject to limitations. Theoretically, there could be an underestimation of mortality for Asian immigrants due to the Salmon Bias, in which a selective migration of immigrants with deteriorated health return to their home country for death [47]. However, this phenomenon is unlikely to be more pronounced among less-educated Asian women; therefore, it would not impact our results. Also, as individual-level menopausal status was unknown, age 50 and above was used as a proxy to identify postmenopausal cases, which may have resulted in some misclassification of PMBC cases. For Chinese and Filipino populations, smaller populations estimated by the American Community Survey may be affected by some degree of sampling variation [48]. Additionally, available individual-level mortality data is limited, devoid of clinical, risk, and prognostic factor data (e.g., obesity, hormone therapy, mammography, etc.) that influence the development of and prognosis for PMBC. Lacking individual level data, we relied on survey data. We also had no specific information on treatment or genetic-related factors.

In conclusion, our study provides novel findings regarding the unique relationship between educational attainment and mortality among postmenopausal Asian women with breast cancer. Higher population-based mortality rates for PMBC among Asian women with higher educational attainment can likely be attributed to differences in the epidemiological profile of risk factors that lead to increased incidence rather than diminished survival. Since US cancer registries unfortunately do not collect data on educational level, our study is important as we show for the first time, to our knowledge, that a high PMBC incidence can be inferred from mortality, given the documented survival advantage among women with higher education. The process of immigration and acculturation among Asian women in the US has important and lasting effects on the health of this population, the fastest-growing minority in the US. Moreover, majority foreign-born populations such as Asians are considerably heterogeneous in many ways, including educational attainment and other associated socio-demographic characteristics that impact cancer patterns. Nonetheless, a decrease in PMBC risk is at least theoretically attainable for higher educated Asian women. Viewing Asians as the stereotypical "model minority" in research, highlighting only their positive cancer outcomes [49], masks important disparities in their breast cancer mortality. Identification of any possibly modifiable breast cancer risk factors among higher educated women, in the context of multiple social and health programs, could lead to better equilibrium across all populations, with a decrease in incidence towards the lowest levels seen for each group. This is an area of research that requires more exploration.

## Supporting information

S1 Table Average prevalence^a^ of PMBC risk factors for NHW and Asian women by education level among age 40 and older; source: 2012–2017, NHIS, West region. (DOCX)
