# Supplementary material for: Elevated breast cancer mortality among highly educated Asian American women
Source: PLoS One. 2022 May 18;17(5):e0268617. doi: 10.1371/journal.pone.0268617 (PMC9116645; doi:10.1371/journal.pone.0268617)
Supplement: S1 Table — (DOCX) [file pone.0268617.s001.docx]

| **Supplementary Table 1. Average prevalence^a^ of PMBC risk factors for NHW and Asian women by education level among age 40 and older; Source: 2012-2017, NHIS, West Region.** | | | | | | | |
| --- | --- | --- | --- | --- | --- | --- | --- |
|  | **HRT**  **% (95% CI)** | **Mammography**  **% (95% CI)** | **Obesity**  **% (95% CI)** | **Parity** | | | |
|  |  |  |  | **Mean Age at Birth of 1^st^ Child** | **Nulliparous**  **% (95% CI)** | **Parous**  **% (95% CI)** | **≥3 Births^b^**  **% (95% CI)** |
| **NHW** |  |  |  |  |  |  |  |
| Less than High School | 12.5 (5.6-25.4) | 85.7 (76.9-91.6) | 35.9 (30.4-41.7) | 19.9 (18.6-21.1) | 6.9 (2.5-17.7) | 93.1 (82.3-97.5) | 50.1 (38.4-61.8) |
| High School or Equivalent | 22.7 (18.3-27.8) | 92.3 (90.6-93.7) | 29.1 (27.3-31.0) | 22.6 (22.0-23.2) | 15.5 (12.7-18.9) | 84.5 (81.1-87.3) | 35.6 (31.1-40.3) |
| Associate's Degree or Higher | 23.9 (19.3-29.2) | 93.7 (92.2-95.0) | 23.1 (21.5-24.8) | 26.5 (25.8-27.3) | 26.8 (22.7-31.4) | 73.2 (68.6-77.3) | 22.7 (19.2-26.6) |
| p for linear trend | 0.037 | 0.013 | <0.0001 | <0.0001 | <0.0001 | <0.0001 | <0.0001 |
| Total | 22.7 (19.4-26.3) | 92.6 (91.4-93.6) | 26.4 (25.2-27.8) | 24.2 (23.7-24.7) | 20.7 (18.3-23.4) | 79.3 (76.6-81.7) | 29.9 (27.2-32.8) |
|  |  |  |  |  |  |  |  |
| **Asian** |  |  |  |  |  |  |  |
| Less than High School | 0.8 (0.1-3.9) | 73.9 (63.4-82.2) | 10.0 (6.5-15.0) | 23.1 (21.2-24.9) | 13.0 (4.5-32.2) | 87.0 (67.8-95.5) | 66.4 (47.8-81.0) |
| High School or Equivalent | 7.5 (3.1-17.2) | 79.6 (70.0-86.7) | 7.8 (5.3-11.4) | 25.3 (23.5-27.1) | 15.6 (8.2-27.4) | 84.4 (72.6-91.8) | 31.3 (19.8-45.6) |
| Associate's Degree or Higher | 9.5 (5.8-15.1) | 89.6 (84.0-93.5) | 8.1 (6.1-10.6) | 28.6 (27.6-29.6) | 26.9 (20.5-34.5) | 73.1 (65.5-79.5) | 13.3 (7.4--22.8) |
| p for linear trend | <0.0001 | 0.002 | 0.694 | <0.0001 | <0.0001 | <0.0001 | <0.0001 |
| Total | 7.9 (5.1-12.2) | 84.9 (81.2-88.1) | 8.3 (6.7-10.2) | 26.8 (26.0-27.7) | 22.1 (16.4-29.1) | 77.9 (70.9-83.6) | - 1. (17.8-33.4) |
| Abbreviation: HRT, Hormone replacement therapy; NHIS, National Health Interview Survey; NHW, Non-Hispanic White; PMBC, Postmenopausal breast cancer.  a. Weighted using the final sample adult weights to incorporate complex survey design; b. Proportion of women with ≥3 births is calculated among all women, both nulliparous and parous. | | | | | | | |
